# Year-Long Metagenomic Study of River Microbiomes Across Land Use and Water Quality

**DOI:** 10.3389/fmicb.2015.01405

**Published:** 2015-12-16

**Authors:** Thea Van Rossum, Michael A. Peabody, Miguel I. Uyaguari-Diaz, Kirby I. Cronin, Michael Chan, Jared R. Slobodan, Matthew J. Nesbitt, Curtis A. Suttle, William W. L. Hsiao, Patrick K. C. Tang, Natalie A. Prystajecky, Fiona S. L. Brinkman

**Affiliations:** ^1^Department of Molecular Biology and Biochemistry, Simon Fraser UniversityBurnaby, BC, Canada; ^2^Department of Pathology and Laboratory Medicine, University of British ColumbiaVancouver, BC, Canada; ^3^British Columbia Public Health Microbiology and Reference Laboratory, British Columbia Centre for Disease ControlVancouver, BC, Canada; ^4^Coastal Genomics Inc., BurnabyBC, Canada; ^5^Department of Microbiology and Immunology, University of British ColumbiaVancouver, BC, Canada; ^6^Department of Earth, Ocean and Atmospheric Sciences, University of British ColumbiaVancouver, BC, Canada; ^7^Department of Botany, University of British ColumbiaVancouver, BC, Canada; ^8^Canadian Institute for Advanced ResearchToronto, ON, Canada

**Keywords:** metagenomics, rivers, freshwater, land use, temporal variation, normalization, bacteria

## Abstract

Select bacteria, such as *Escherichia coli* or coliforms, have been widely used as sentinels of low water quality; however, there are concerns regarding their predictive accuracy for the protection of human and environmental health. To develop improved monitoring systems, a greater understanding of bacterial community structure, function, and variability across time is required in the context of different pollution types, such as agricultural and urban contamination. Here, we present a year-long survey of free-living bacterial DNA collected from seven sites along rivers in three watersheds with varying land use in Southwestern Canada. This is the first study to examine the bacterial metagenome in flowing freshwater (lotic) environments over such a time span, providing an opportunity to describe bacterial community variability as a function of land use and environmental conditions. Characteristics of the metagenomic data, such as sequence composition and average genome size (AGS), vary with sampling site, environmental conditions, and water chemistry. For example, AGS was correlated with hours of daylight in the agricultural watershed and, across the agriculturally and urban-affected sites, k-mer composition clustering corresponded to nutrient concentrations. In addition to indicating a community shift, this change in AGS has implications in terms of the normalization strategies required, and considerations surrounding such strategies in general are discussed. When comparing abundances of gene functional groups between high- and low-quality water samples collected from an agricultural area, the latter had a higher abundance of nutrient metabolism and bacteriophage groups, possibly reflecting an increase in agricultural runoff. This work presents a valuable dataset representing a year of monthly sampling across watersheds and an analysis targeted at establishing a foundational understanding of how bacterial lotic communities vary across time and land use. The results provide important context for future studies, including further analyses of watershed ecosystem health, and the identification and development of biomarkers for improved water quality monitoring systems.

## Introduction

High quality freshwater is an essential natural resource and is increasingly threatened by human activities (Vörösmarty et al., 2010). Microbes have long been used as sentinels of poor water quality due to their potential to be host specific, which enables source tracking, and/or their sensitivity to changes in the environment (White and Wilson, 1989; White et al., 1998). For example, *Escherichia coli* or coliform bacterial abundance is widely used as a proxy to monitor fecal contamination in drinking and recreational water. However, these tests rely on culturing under selective conditions and are susceptible to both false positive and false negative results (Goldstein et al., 1996; McLain et al., 2011). In clinical settings, pathogen-specific diagnostics have moved toward PCR-based tests due to increased sensitivity and specificity and decreased turnaround times (Espy et al., 2006). A similar methodological shift has begun in environmental monitoring for human health (e.g., United States Environmental Protection Agency Method 1609 to test for Enterococci with qPCR); however, effective biomarkers of ecosystem health have yet to be developed. Recent studies have identified taxonomic ratios associated with fecal contamination (Garrido et al., 2014; Li et al., 2015) and PCR-based tests for fecal indicator bacteria have been developed, yet these still suffer the weaknesses of being either broadly distributed but susceptible to false positives or are highly specific but susceptible to false negatives (van der Wielen and Medema, 2010; Harwood et al., 2013). Further, these tests focus on fecal contamination, which is valuable yet insufficient to monitor the health of an aquatic ecosystem (Palmer and Febria, 2012). Rivers are the chief source of renewable water for humans and freshwater ecosystems (Vörösmarty et al., 2010), yet microbial diversity in lotic (i.e., flowing water) communities is less commonly studied than in marine or lake ecosystems, and among those studied, contaminated systems are underrepresented (Zinger et al., 2012). In order to develop better tests for ecosystem health, foundational data is required to answer fundamental questions about lotic microbiota such as how they are affected by land use and how they vary over time.

Microbial communities can be described in terms of levels of diversity (e.g., richness, evenness) and composition (which taxa and genes are present). Assessing the former has been possible for decades, and the importance of characterizing microbial communities is reflected in the hundreds of studies that have investigated how levels of diversity are affected by environmental changes (Zeglin, 2015). However, these studies have been limited to describing community diversity, instead of composition, due to the technologies available at the time. While important for biological understanding, metrics of diversity are difficult to translate into diagnostics. With the development of microbiome studies based on DNA sequencing, the effect of environmental conditions on microbial community composition has begun to be revealed. For example, using taxonomic characterization of riverine bacterial microbiomes, studies have shown that bacterioplankton communities vary by location and nutrient concentrations (Hu et al., 2014; Jackson et al., 2014; Read et al., 2014; Ruiz-González et al., 2015; Savio et al., 2015; Wang et al., 2015); however, these studies were limited by the duration of the sampling periods, which were less than a month, with most sites sampled only once. In a 3-year study of bacterioplankton community composition, annual community reassembly was observed and seasonal shifts were inferred, though this study focused on spring and summer, with only two samples collected in the fall and none in the winter (Fortunato et al., 2013). Seasonal taxonomic shifts could be important in predicting the effect of contamination on microbial communities. For example, a study that sampled an urban river once per season for one year found variability in the recovery of bacterial community composition after exposure to sewage eﬄuent (García-Armisen et al., 2014). Further investigating anthropogenic contamination, another study, limited to the early summer over two years, indicated that land use affected the taxonomic composition of bacterial communities in a river across forested, urban and agricultural sites (Staley et al., 2014a).

Beyond taxonomic characterization, two studies have described the metagenomes (total genetic material) present in lotic free-living microbial communities. The first study looked at one sample from the pristine upper course of the Amazon River and saw an overrepresentation of pathways involved in heterotrophic carbon processing, especially from plant material, as compared with marine samples (Ghai et al., 2011). The second looked at 11 sites in the Upper Mississippi River watershed with near-by land use characterized as forested, urban or agricultural (Staley et al., 2014b). In this research, the authors found that the core functional traits were largely stable across sampling sites; however, this study was based on one sample from each site and did not examine potential land use effects over time.

We hypothesized that metagenome characteristics and functional traits vary across sampling sites in lotic bacterial communities, but that this variability may require sampling across time to better observe and characterize them. Here, we present a year-long survey of bacterial genes in sampling sites across rivers in protected, agricultural, and urban watersheds. We found that metagenomic characteristics, as well as abundance of gene functional groups vary with time, land use and environmental conditions. Further, we discuss trends in average genome size (AGS), which have been shown to be important for analysis of gene functions (Manor and Borenstein, 2015; Nayfach and Pollard, 2015), and demonstrate how normalization techniques are important to consider in these analyses. The data presented in this study is a valuable resource for the study of bacterial lotic communities. The analyses presented here aim to provide a foundational interpretation of this data that contributes to the understanding of the variability of lotic bacterial microbiomes over time and land use. This work will provide support for future developments in water quality monitoring, both directly from the sequence data presented, which could be used to identify DNA sequence biomarkers indicative of land use or water quality conditions, and indirectly by providing context aiding design of future water quality studies.

## Materials and Methods

### Sample Collection, DNA Sequencing, and Environmental Measurements

Monthly samples of flowing freshwater were collected from seven sites in three watersheds up to 130 km apart in Southwestern British Columbia, Canada. Some of the sites were pristine (protected from land use) while others were affected by agricultural or urban activity (**Table 1**). Watersheds were sampled monthly on different days. All sites within a watershed were sampled on the same day within two and a half hours. Twelve samples were collected from the urban watershed and 13 were collected from both the protected and agricultural watersheds. All rivers were less than 10 m wide at the sampling location except at site ADS, which was 30 m wide. Near-surface water was collected from rivers at all sites except PDS, where water was collected after residence in a reservoir and passing through a pipe. PDS is the only site downstream of a lake or reservoir.

**Table 1:** Description of sampling sites across watersheds with varying land use.

Watershed	Site name	Catchment land use	Description
Agricultural	AUP (Agri-Upstream)	Forest and minimal housing	Upstream of agricultural “pollution”. Not affected by agricultural activity. Collected from a small rocky stream near the base of a forested hill with minimal housing nearby.
	APL (Agri-Pollution)	Agriculture	At site of agricultural “pollution”. Collected from a slough in an intensely farmed and irrigated floodplain with minimal tree cover. AUP is upstream of floodplain, separated by 9 km.
	ADS (Agri-Downstream)	Agriculture and some urban	Downstream of agricultural “pollution”. Collected from a river fed by an agricultural floodplain (site of APL) as well as a separate tributary from a more distant agricultural and urban area. Minimal tree cover throughout catchment. ADS is 2.5 km from APL.
Urban	UPL (Urban-Pollution)	Forest and urban	At site of urban “pollution”. Collected from a stream that originates in mountainous forest then passes through 300 m of residential development.
	UDS (Urban-Downstream)	Forest, parks, and urban	Downstream of urban “pollution”. Collected downstream of UPL, after passing through 1 km of residential neighborhood (half houses and half treed parks).
Protected	PUP (Protected)	Forest	Collected from river in forested, protected watershed that feeds a drinking water reservoir. Collected 1 km upstream of entry point to reservoir.
	PDS (Protected-Downstream)	Forest, reservoir, and pipe	Downstream of PUP-fed reservoir, which is 1 km wide by 7 km long. Sample collected after reservoir water has passed through a 9 km long pipe, 2 m in diameter. Water enters pipe from reservoir on the opposite side of reservoir from PUP. PDS is 16 km from PUP.

At each sampling event, 40 L of water were collected, prefiltered through a 105-μm pore-size spectra/mesh polypropylene filter (SpectrumLabs, Rancho Dominguez, CA, USA) to remove larger particles then transported on ice to the lab for further processing within 24 h. Water was filtered sequentially through 1 μm pore-size filter (Envirochek HV, Pall Corporation, Ann Harbor, MI, USA) then a 0.2 μm pore-size filter (142 mm Supor-200 membrane disk filter, Pall Corporation, Ann Harbor, MI, USA) to collect bacterial and archaeal sized cells. This pre-filtration did also remove larger and particle-associated bacterial cells, and of course bacteria adhering to large items like leaves. Cells were collected off the 0.2 μm pore-size filters by vortexing with tungsten beads (i.e., bead beating) and centrifugation. DNA was extracted using the PowerLyzer Powersoil DNA Isolation Kit (Mo Bio, Carlsbad, CA, USA). Cells were mainly bacterial and not archaeal (data not shown), so for simplicity, the microbial community is here referred to as bacterial.

A positive control (mock community) was prepared by spiking de-ionized water with DNA extracted (NucleoSpin Tissue, Macherey-Nagel, Düren, Germany) from 12 cultured bacterial strains [*Bacillus amyloliquefaciens* FZB42, *Bacillus cereus* ATCC 14579, *Burkholderia cenocepacia* J2315, *Escherichia coli* K-12, *Frankia* sp. CcI3, *Micrococcus luteus* NCTC 2665, *Pseudomonas aeruginosa* PAO1, *Pseudomonas aeruginosa* UCBPP-PA14, *Pseudomonas fluorescens*, Pf-5, *Pseudomonas putida* KT2440, *Rhodobacter capsulatus* SB 1003, *Streptomyces coelicolor* A3(2)]. Ultrapure (Type 1) water (Milli-Q, Millipore Corporation, Billerica, MA, USA) was used as a negative control.

Shotgun sequencing libraries were prepared using the Nextera XT DNA sample preparation kit (Illumina, Inc., San Diego, CA, USA). Gel-size selection was automated with Ranger Technology (Coastal Genomics Inc., Burnaby, BC, USA) to ensure consistent and specific fragment lengths, targeting 500–800 bp (Uyaguari-Diaz et al., 2015). Sequencing was performed using a MiSeq platform (Illumina, Inc., San Diego, CA, USA) using the MiSeq Reagent Kit V2 (2× 250 bp paired-end reads, 500 cycles) at the British Columbia Public Health Microbiology and Reference Laboratory. Samples were sequenced over seven runs and a positive and negative control sample was included in each run. All raw sequences are deposited in the NCBI Sequence Read Archive under BioProject ID: 287840.

Physical water quality parameters were measured *in situ* using a YSI Professional Plus handheld multiparameter instrument (YSI Inc., Yellow Springs, 96 OH) and VWR turbidity meter (model 66120-200, VWR, Radnor, PA, USA). Chemical parameters were measured as follows: dissolved chloride using an automated colorimeter (SM-4500-Cl G), ammonia using phenate methods (SM-4500-NH3 G; Eaton and Franson, 2005), orthophosphates using chemical precipitation (Murphy and Riley, 1962), and nitrites and nitrates using spectrometry (Wood et al., 1967). Chlorophyll *a* was measured using fluorometric analysis (Welschmeyer, 1994). Abundances of bacterial size particles were estimated using a FACSCalibur flow cytometer (Beckton Dickinson, San Jose, CA, USA) with a 15 mW 488 nm air-cooled argon-ion laser (Brussaard et al., 1999) followed by analysis using CYTOWIN version 4.31 (2004), (Vaulot, 1989). Precipitation records and hours of daylight were collected from the Canadian Climate Data database (Environment Canada, 2013), from the closest station per watershed (station IDs: agricultural 1100031, urban 1105669, protected 1017230). Missing rainfall values were replaced with the mean value across 2 days before and 2 days after sampling. Rainfall values used for analysis are cumulative over 3 days prior to sampling. Minimal snowfall occurred. Other missing values were replaced with sampling site means. The Canadian Council of Ministers of the Environment’s Water Quality Index was calculated using their provided spreadsheet (Canadian Council of Ministers of the Environment, 2007) based on guidelines for ammonia, chloride, DO, nitrate, pH, and phosphorous.

### Statistical Analyses

All statistical analyses were performed using R 3.2.0, including methods from the Vegan package (Oksanen et al., 2015). Reported means and medians are followed by the standard deviation, proceeded by the “±” symbol. Correlations were assessed using Spearman correlation coefficients, unless otherwise noted. Where applicable, *p*-values were corrected for false discovery rate using the Benjamini and Hochberg (1995) procedure. Significance test values were considered significant if *p*-values were less than 0.05 and *q*-values were less than 0.1.

### Metagenome Compositional Analysis

Shotgun sequenced reads were trimmed to remove low quality bases using Trimmomatic (Bolger et al., 2014). A sliding window of length 5 and a minimum Phred score of 20 was used at the 3′ end. At the 5′ end, sequences of one or more nucleotides with scores less than 20 were trimmed. Sequencing adapters were removed using Cutadapt (Martin, 2011), overlapping paired-end reads were merged using PEAR (Zhang et al., 2014), and reads shorter than 100 bp were discarded. After this processing, samples had 418538 to 2165162 high quality reads and samples were subsampled to the number of reads in the smallest sample: 418500 reads. The January sample from site PUP was omitted from all analyses due to too few reads.

Environmental conditions associated with the samples were compared based on scaled measures using Euclidean distances, clustered using Ward’s method (R: ward.D2), and visualized using non-metric multidimensional scaling (NMDS) with two axes. Hierarchical clustering was performed and evaluated using the pvclust R package (Suzuki and Shimodaira, 2006). Bootstrap *p*-values were based on multiscale bootstrap resampling with 10000 repetitions.

The k-mer abundance profile of each sample was calculated by counting the frequencies all nucleotide sequences (k-mers) of length 12 in each dataset using Jellyfish (Marçais and Kingsford, 2011). These k-mer profiles were compared using Manhattan distances, as appropriate for high dimensional datasets (Aggarwal et al., 2001), clustered using Ward’s method (R: ward.D2) and visualized using hierarchical clustering and NMDS with two axes. Major sample clustering patterns discussed below were consistent with k-mer lengths 4, 9, and 10 (data not shown), but were most distinct with higher k-mer lengths.

### Calculation and Normalization of Functional Gene Group Abundances

Reads were compared against NCBI’s nr database (downloaded April 9, 2014) using RAPSearch2 (Zhao et al., 2012). Resulting protein alignments longer than 30 amino acids were analyzed using MEGAN version 5.10 (Huson et al., 2011) with default parameters, including a minimum bit score of 35 and a maximum *e*-value of 0.01, to determine the gene families present, using the SEED (Overbeek et al., 2005) and KEGG gene functional group databases (Kanehisa, 2000). Gene group abundance profiles were analyzed for differential abundance using the Wilcox test after removing low abundance features (mean abundance <0.01% in all samples). Subsamples of 100,000 reads were also analyzed using MG-RAST (Meyer et al., 2008) with default parameters for comparative purposes.

The quality of the MEGAN assignments of reads to gene groups was assessed using the mock community samples. These samples of known taxonomic composition were annotated using MEGAN and the KEGG database of ortholog groups. These mock community KEGG profiles were compared against reference profiles, compiled from annotated genomes. The KEGG database was used for this analysis due to the availability of annotated genomes, however, the version of the KEGG database used for this reference annotation was not the same as the version used in the MEGAN analysis. Ortholog groups missing from one of the two profiles under comparison, possibly due to differences in database version, were omitted from this analysis, leaving 1725 KEGG ortholog groups to compare. Annotation profiles were fairly well correlated between the MEGAN and reference datasets when looking at KEGG ortholog groups (*r* = 0.74, *p* < 2.2*e*–16, Pearson correlation used due to interest in linear relationship). This correlation improved when looking at KEGG pathways (*r* = 0.96, *p* < 2.2*e*–16). Of the 208 pathways, two in particular were predicted as less abundant in the MEGAN profiles relative to the reference profiles: “Ribosome” and “ABC transporter”. When these pathways were removed, the correlation rose to *r* = 0.98. Adjusting abundance profiles by the average KEGG ortholog group gene length improved the correlations between ortholog group profiles (*r* = 0.86, *p* < 2.2*e*–16) but the improvement was minimal for pathway profiles (*r* = 0.99, *p* < 2.2*e*–16).

Normalization of gene functional group abundance profiles by AGS was performed on subsampled reads with two approaches. The first used MicrobeCensus to estimate AGS values (Nayfach and Pollard, 2015), which were then divided by the mean AGS across samples (to avoid inconveniently large numbers) and then multiplied by group abundances; the second used MUSiCC, which adjusts group abundances directly (Manor and Borenstein, 2015). Both tools are based on the same goal: to calculate normalization factors such that normalized universal, single copy gene abundances will be constant across samples. These tools assume that all reads are bacterial and so can be affected by the presence of eukaryotic DNA sequences. Due to the filtration strategy used during sample processing, very little eukaryotic DNA was present in the samples (median 2 ± 0.7% of domain-assigned reads). Both tools gave very similar results, with an overall Pearson correlation of 0.998 (*p* < 2.2*e*–16) between KEGG ortholog group abundance profiles across all samples, and a correlation score of 0.997 (*p*-values < 2.2*e*–16) within each sample. Currently, MUSiCC only accommodates KEGG and COG profiles and normalizes assigned reads, whereas MicrobeCensus works directly on reads to estimate AGS and therefore allows the flexibility of using any downstream functional assignment tool. In the analyses that follow, MicrobeCensus normalization is used.

## Results and Discussion

### Contamination and Water Chemistry is Reflected in Reference-Free Clustering of Metagenomes Across Land Use

**FIGURE 1:**
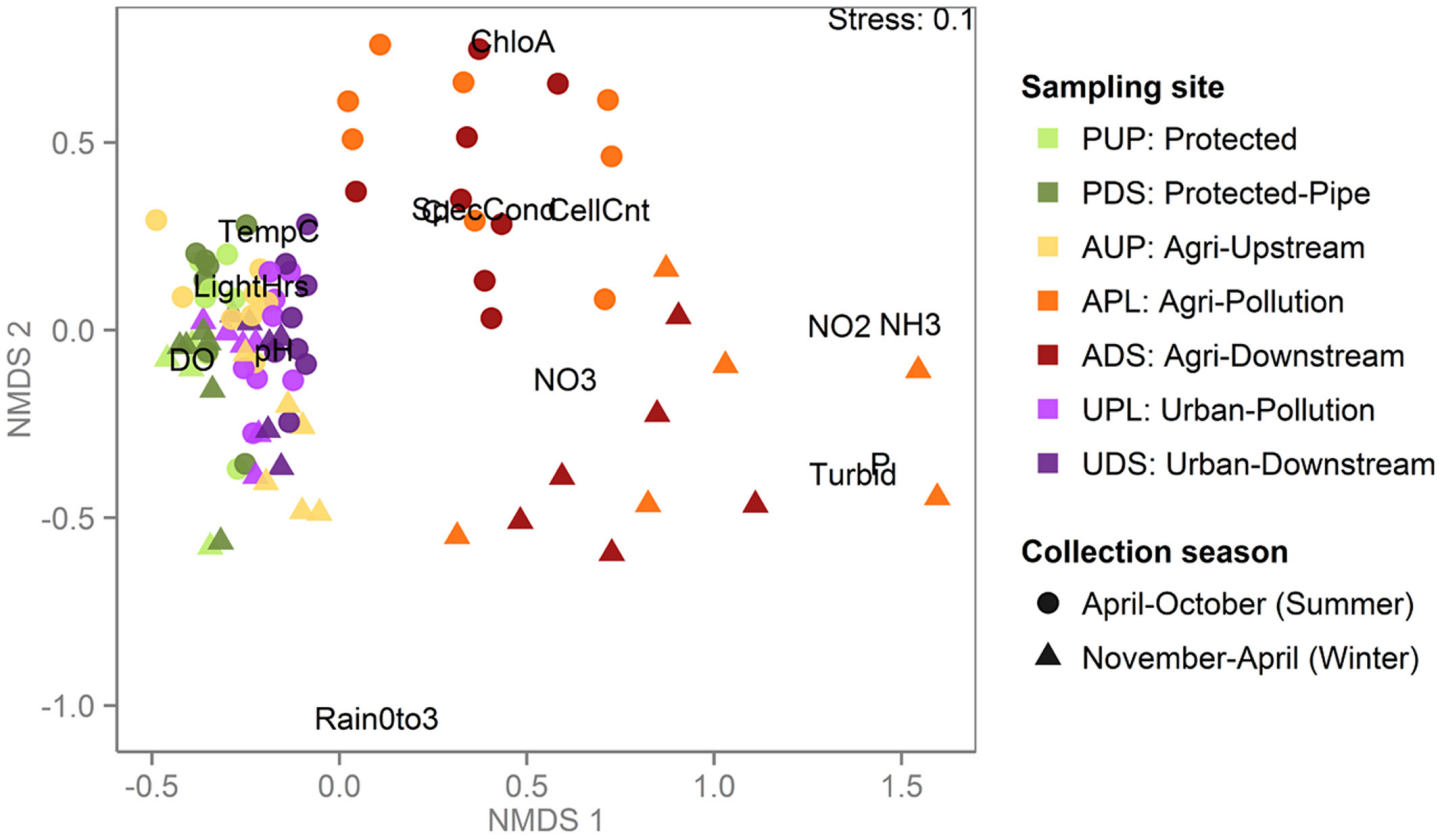
**Agriculturally affected sites are distinct from protected and urban sites when clustered by water chemistry and environmental variables**. Non-metric multidimensional scaling (NMDS) plot based on environmental and chemical water measurements in which each point represents a sample, colored by sampling site and shaped by season during which the sample was collected. Environmental measures are abbreviated as in **Table 2**. Samples from the agriculturally affected sites (APL and ADS) are most distinct, with summer and winter samples mostly clustering together, reflecting the winter’s increased rain and consequent agricultural runoff. All other samples are more similar to each other than to the agriculturally affected sites, including the samples from the agricultural watershed that were collected upstream of agricultural activity (AUP).

Metagenomic shotgun sequencing of free-living bacterial communities was performed on 89 samples, collected monthly from seven sites across three watersheds under varying land use (protected, urban or agricultural; **Table 1**). The agriculturally affected sites (APL and ADS) had the highest concentrations of nutrients and were the most distinct in terms of water chemistry, while the urban affected sites (UPL and UDS) and the unaffected sites (PUP and AUP) were more similar and had higher concentrations of dissolved oxygen (**Table 2** and **Figure 1**).

**Table 2:** Summary of environmental variables over 1 year of sampling: means and standard deviations.

		Agricultural			Urban		Protected	
Measured variable	Abbreviation	AUP	APL	ADS	UPL	UDS	PUP	PDS
**Water properties**								
Ammonia mg/L	NH3	0.03 ± 0.08	0.52 ± 0.36	0.16 ± 0.11	0.01 ± 0.01	0.02 ± 0.01	0.01 ± 0	0.02 ± 0.03
Dissolved chloride mg/L	Cl	1.9 ± 2.9	14.9 ± 9	10.7 ± 3.7	6.2 ± 3.3	10.0 ± 3.7	2.1 ± 0.5	2.6 ± 0.3
Dissolved oxygen mg/L	DO	15 ± 5	5 ± 3.8	10 ± 3.4	11.7 ± 2.5	11.6 ± 2	11.5 ± 1.4	10.7 ± 1.8
Nitrite mg/L	NO3	2.8 ± 0.5	4.4 ± 3.6	8.4 ± 1.3	2.8 ± 1	2.8 ± 1	0.2 ± 0.2	0.09 ± 0.04
Nitrite mg/L	NO2	0.01 ± 0	0.2 ± 0.2	0.1 ± 0.07	0.01 ± 0.01	0.02 ± 0.01	0.02 ± 0.06	0.01 ± 0.01
pH	pH	7.2 ± 0.3	6.9 ± 0.3	7.3 ± 0.2	6.4 ± 0.4	7.0 ± 0.3	6.6 ± 0.7	6.8 ± 0.4
Orthophosphate mg/L	P	ND	0.11 ± 0.11	0.13 ± 0.11	0.01 ± 0.01	0.01 ± 0	ND	ND
Specific Conductivity uS/cm	SpecCond	106 ± 23	305 ± 31	266 ± 51	75 ± 28	105 ± 26	77 ± 35	52 ± 21
Turbidity NTU	Turbid	2.3 ± 5	19 ± 11	19 ± 15	0.8 ± 0.5	1.8 ± 1.2	0.3 ± 0.1	0.4 ± 0.1
Temperature °C	TempC	8 ± 3	12 ± 5	12 ± 5	9 ± 4	9 ± 4	8 ± 5	10 ± 4
**Biological measures**								
Chlorophyll *a* μg/L	ChloA	0.4 ± 0.4	2 ± 2	2 ± 2.3	0.1 ± 0.1	0.6 ± 1.2	0.1 ± 0.05	1 ± 0.5
Flow cytometry million cells/mL	CellCnt	0.4 ± 0.7	2 ± 1.5	1.8 ± 1.3	0.3 ± 0.2	0.3 ± 0.2	0.2 ± 0.4	0.5 ± 0.6
**Environmental measures**								
Rainfall mm	Rain0to3	22 ± 24	22 ± 24	22 ± 24	19 ± 19	19 ± 19	14 ± 23	14 ± 23
Daylight hours	LightHrs	13.6 ± 2.7	13.6 ± 2.7	13.6 ± 2.7	13.5 ± 2.8	13.5 ± 2.8	13.5 ± 2.8	13.5 ± 2.8

*ND (non-detect): value was below detection limit*.

**FIGURE 2:**
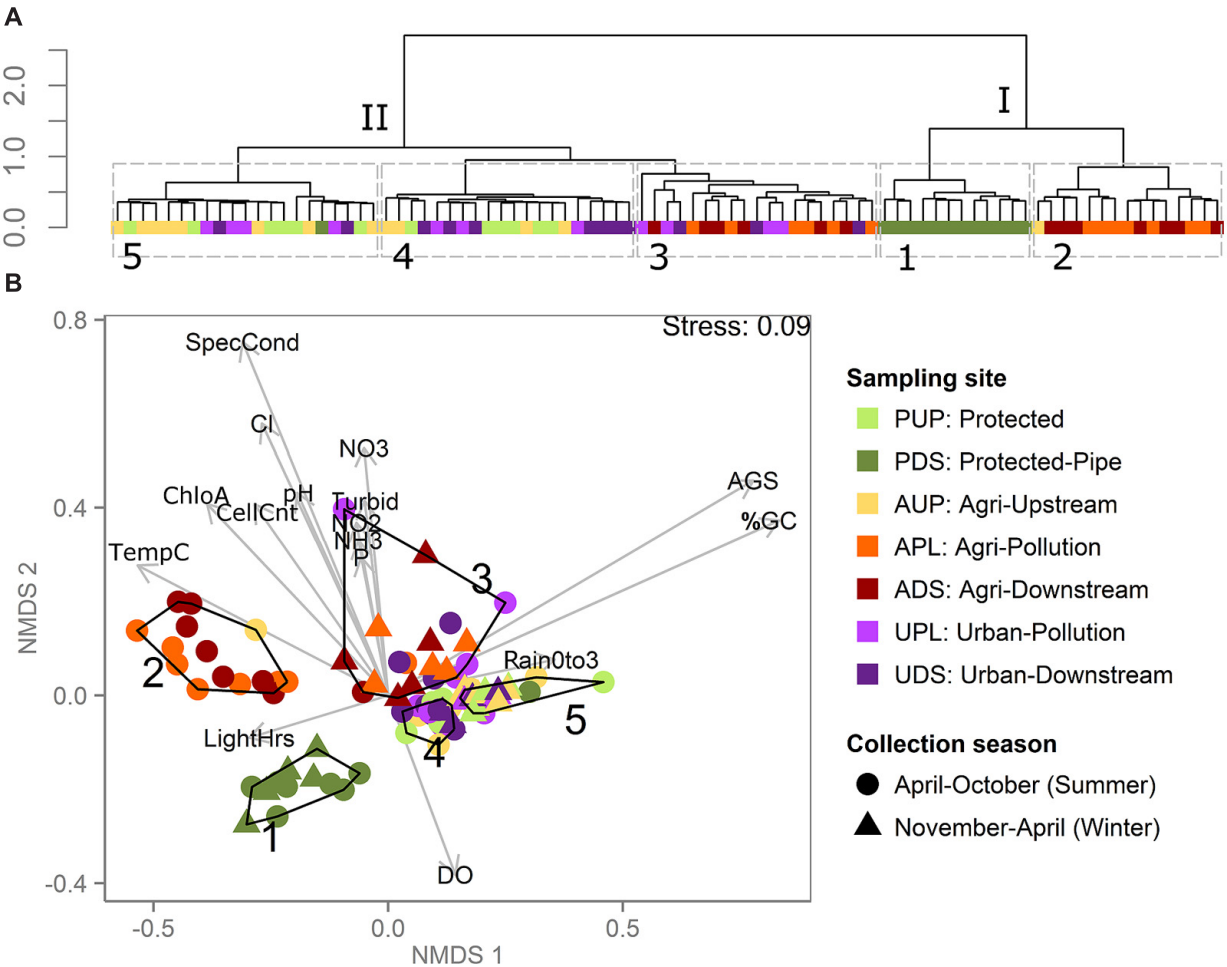
**Metagenomes clustered by reference-free k-mer analysis show effect of sampling site, weather conditions, and water chemistry. (A)** Hierarchical clustering of samples based on metagenome k-mer composition. Each terminal node (leaf) is a sample, colored by sampling site. Roman numerals label major clusters, Arabic numerals label sub-clusters outlined in gray dashed lines. **(B)** NMDS plot based on k-mer abundance distributions in which each point represents a sample, colored by sampling site. Clusters outlined and numbered in black correspond to numbered clusters in **(A)**. Environmental variables were correlated with ordination axes using envfit and are displayed using gray arrows, where lengths of arrows correspond to the strength of the correlation between the variable and the ordination (only variables with *p* < 0.05 displayed) and direction corresponds to increasing value (e.g., samples closer to the bottom of the plot have higher DO). Environmental measures are abbreviated as in **Table 2**. The percentage of nucleotides that are G or C is abbreviated as “%GC”. Sampling site is the major distinction among metagenomes from flowing surface water versus water collected from a reservoir-fed pipe (PDS). Among surface water samples, clustering reflects samples’ collection date, water chemistry and land use.

Clustering metagenomes by the abundance of constant-length DNA sub-sequences (k-mers) has been shown to be an effective way to characterize microbiomes without the biases or limitations of existing microbial references (Jiang et al., 2012; Hurwitz et al., 2014). Here, clustering river metagenomes based on k-mer abundance resolves samples into clusters that share common sampling sites, watersheds, or environmental conditions (**Figure 2A**). Using hierarchical clustering, we observe two major clusters, I and II, that each divide into two and three smaller clusters, respectively. Cluster I is composed of two sub-clusters: Cluster 1, which is composed of 12 of 13 samples from the post-pipe (PDS) site, and Cluster 2, which is composed of a subset of samples collected from the agriculturally affected sites (APL, ADS). Cluster II is composed of three sub-clusters: Clusters 4 and 5, which mostly contain samples from the unaffected (AUP, PUP) and urban sites (UPL, UDS), and Cluster 3, which contains samples both from the agriculturally affected sites and from the urban sites.

Though the PDS site is similar to the others in this study in terms of the water quality parameters measured (**Figure 1**), it stands apart in terms of its metagenomes’ k-mer compositions (**Figure 2A**). This is likely due to how different this sampling site is from the rest in this study: PDS samples were collected from a reservoir after passing through a 9-km pipe, while all other samples were collected from near-surface water from rivers. The microbiota of these samples were likely significantly affected by both residency in the reservoir (Crump et al., 2012) and transmission through the pipe (Fisher et al., 2015). The clustering pattern described above shows that when metagenomes come from very distinct lotic freshwater environments, this difference can be reflected in k-mer abundances. When samples are more similar, however, such as surface water from rivers, k-mer clustering is not simply reflective of sampling site or watershed. In this case, metagenomes cluster by water properties even when collected from unconnected watersheds 130 km apart. This suggests that there is a signal in the k-mer data that can distinguish between unaffected samples (AUP, PUP) and agriculturally affected samples (APL, ADS), but not between unaffected samples and all urban samples (UPL, UDS). This may be because of variability in the impact of urban land use on the rivers. In order to interpret the k-mer clusters in the context of the environmental data collected, we plotted the k-mer distance matrix using NMDS and fit environmental and metadata vectors onto this ordination using envfit (**Figure 2B**). Because k-mer abundances are based on DNA sequence, one major driver of k-mer abundance differences can be nucleotide bias. From **Figure 2B**, we can see that both %GC and AGS tend to have higher values in Clusters 3, 4, and 5 than in Clusters 1 and 2. Nucleotide bias in bacteria has been associated with genome size either as a direct correlation (Musto et al., 2006) or as a more complex association (Mitchell, 2007; Guo et al., 2009). Nucleotide bias has also been shown to be dependent on environmental conditions, independent of shifts in phylogenetic composition (Reichenberger et al., 2015). Here, we see a strong positive linear correlation between AGS and %GC (Pearson’s *r* = 0.8, *p* < 2.2*e*–16).

All agriculturally affected samples are split between two k-mer clusters, which correspond to their sampling period. At these sites, we see two main periods in the year instead of four seasons: rainier “winters” and dryer “summers”, both extending into spring and fall. This is consistent with the general climate of the region. The drier “summer” months were May to October, with an average of 4 ± 4 mm cumulative rainfall over 3 days before sampling, and the “winter” months were November to April, with an average of 43 ± 19 mm cumulative rainfall (*t*-test significant difference *p* = 6*e*–08). Cluster 2 contains samples collected during the drier “summer” months (May to October) and Cluster 3 contains samples collected during the rainier “winter” months (November to April), as well as seven samples from the urban sites collected from May to September. The samples in Cluster 3 tend to have higher values of specific conductivity, pH, turbidity, and cell counts and higher concentrations of orthophosphate, nitrate, nitrite, ammonia, chloride and chlorophyll *a*. Clusters 4 and 5 are associated with higher dissolved oxygen and contain almost all samples (24/25) from the unaffected sites (AUP and PUP) and no samples from the agriculturally affected sites (APL and ADS).

The urban samples are spread among clusters with unaffected (Clusters 4 and 5) and agriculturally affected samples (Cluster 3). The samples that cluster with the high-nutrient, “winter” agriculturally affected samples (Cluster 3) have significantly higher concentrations of orthophosphate than the urban samples from the other two clusters (ANOVA, *p* = 5*e*–05, *q* = 0.0003, means: 0.017 ± 0.005, 0.0082 ± 0.002, 0.0082 ± 0.003, for urban samples in Clusters 3, 4, and 5, respectively). These concentrations are lower than the agriculturally affected samples from this cluster (mean 0.2 ± 0.12), but are close to the water quality guideline limits (0.01–0.02 mg/L depending on season; Canadian Council of Ministers of the Environment, 2007), indicating that this difference in concentration could affect aquatic life. This suggests that the concentration of orthophosphate may have a consistent effect on lotic bacterial communities across watersheds and land use, as previously observed within watersheds (Sterner et al., 2004; Wang et al., 2015) and among photosynthetic biota across habitats (Elser et al., 2007), and that this effect can be detected based on reference-free metagenome analysis.

This clustering technique also highlights potentially unusual samples that do not cluster according to the major trends described above, such as the AUP sample from September, which is the only AUP sample not in Clusters 4 or 5, and the PDS sample from October, which is the only PDS sample not in Cluster 1. Because these samples are very similar to the other samples from their sites in terms of environmental conditions and cell count (**Figure 1**), it suggests that the samples may have been mixed-up or mislabeled during sample processing. Though we cannot be completely certain that these samples are compromised, this possibility is further supported by the unusual AGS of these samples described in the next section; hence, these samples are not included in further analyses.

These results show that in some cases, metagenomes from different sampling sites are distinct despite changing conditions, while in other cases, k-mer clustering reflects variability in samples’ water chemistry and environmental conditions across sampling sites. This analysis demonstrates that there is a bacterial signal that can distinguish between samples collected from an area with agricultural activity versus unaffected samples, but that this signal differs by time of year. Further sampling across multiple years would be required to assess whether this trend is seasonal. While k-mer profiles themselves do not directly translate to efficient water quality tests, this analysis supports that sampling over a year is important in some cases for the development of water quality tests based on bacterial communities. This k-mer analysis is a reference-independent method that reveals that there are land-use, water chemistry, and environmental condition signals in this bacterial metagenomic data.

### Average Genome Size (AGS) Varies with Daylight Hours in the Agricultural Watershed – Illustrating the Importance of Normalization Strategies

**FIGURE 3:**
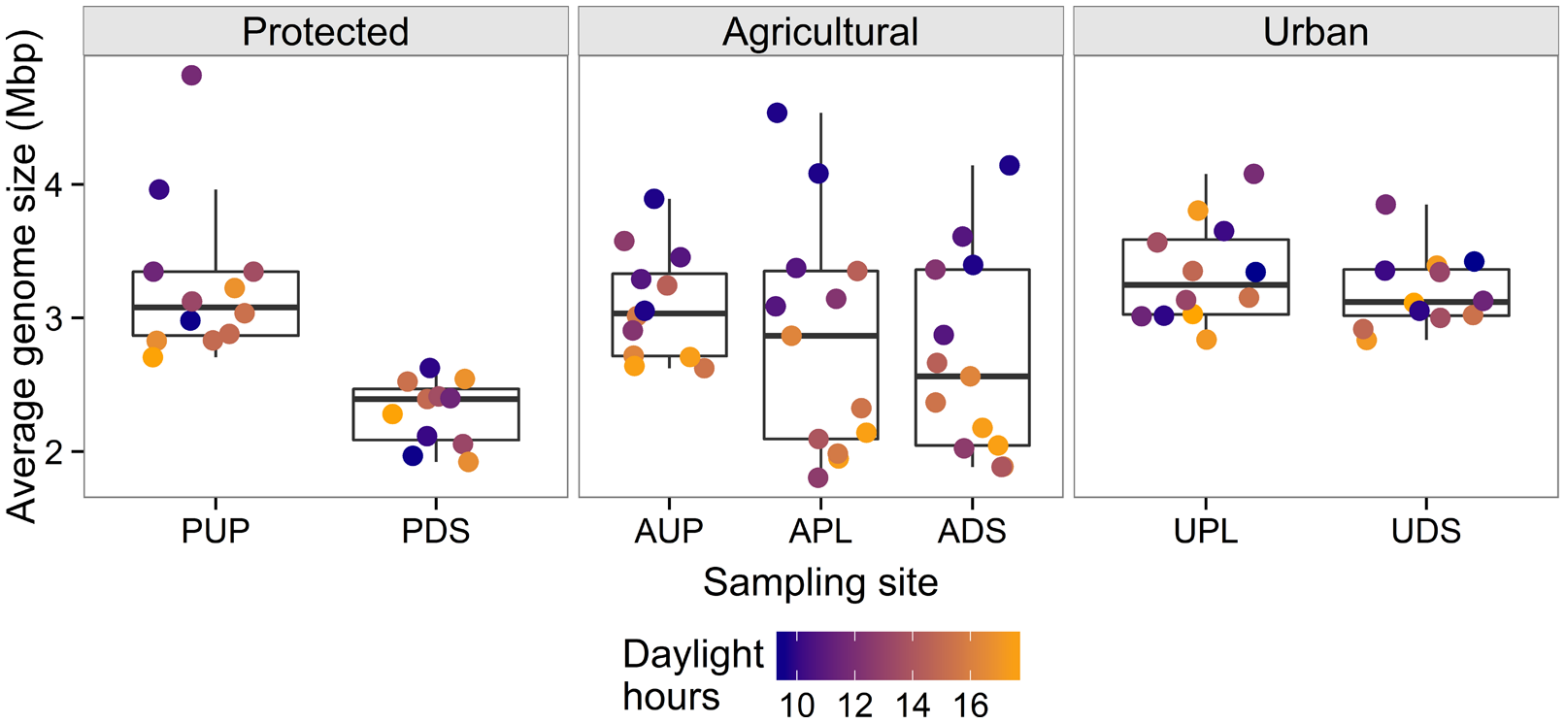
**Average genome size (AGS) across sampling sites, colored by daylight hours, illustrating correlations within the agricultural watershed**. Points represent samples and are jittered on the *x*-axis for visibility. The agriculturally affected sites (APL, ADS) have the largest ranges of values. There is a significant negative correlation between AGS and daylight hours in all sites in the agricultural watershed and a corresponding trend in PUP.

When AGS was tested for correlations with environmental variables from each site (excluding PDS), significant relationships were seen in the agricultural watershed (**Table 3**). At all sites in the agricultural watershed, AGS was significantly negatively correlated with daylight hours. This trend was also seen in the protected site, though was not significant after correcting for multiple testing (PUP: *r* = –0.59; *p* = 0.04; *q* = 0.15) and was not seen in the urban sites (**Figure 3**). These differences among sites may be due to bacterial community differences and/or differences in the effect of sunlight due to variability in the penetrance of light into the water and shade cover (**Table 1**). In the agriculturally affected sites, AGS was also significantly correlated with water temperature, rainfall, and turbidity. The increase in rainfall in both agriculturally affected sites is correlated with increases in turbidity (*r* = 0.68, 0.63; *p* = 0.007, 0.02 for APL and ADS, respectively), consistent with increased rainfall creating runoff from adjacent land, largely agricultural fields. Other potential indicators of runoff from agricultural activity, such as elevated concentrations of orthophosphate, ammonia, dissolved chloride and nitrite are also correlated with AGS (**Table 3**). These relationships indicate that in the agricultural watershed, as day length decreases, AGS increases, and that seasonal rain-associated water chemistry changes also correlate with this change in AGS. Due to the many possible indirect impacts of varying day length, further study would be required to identify the specific environmental drivers of AGS variation. Further sampling would also be required to determine whether this trend is seasonal across years and generalizes to other geographic regions. If it does, this further demonstrates the importance of sampling across time when studying water quality, such as to develop new tests, as signals indicative of agricultural impact may vary substantially over time.

**Table 3:** Environmental conditions significantly correlated with average genome size (AGS) within sampling sites over a year of monthly sampling.

Environmental variable	Site	Spearman’s *rho*	*p*-value	*q*-value (FDR corrected)
Ammonia	ADS	0.63	0.021	0.089
Dissolved chloride	APL	-0.66	0.015	0.073
Dissolved chloride	ADS	-0.75	0.0031	0.03
Nitrite	ADS	0.66	0.015	0.073
pH	APL	-0.8	9.70E-04	0.013
pH	ADS	-0.89	5.60E-05	0.0015
Orthophosphate	APL	0.84	3.20E-04	0.0062
Orthophosphate	ADS	0.64	0.019	0.088
Rainfall	APL	0.78	0.0015	0.017
Rainfall	ADS	0.69	0.009	0.058
Specific conductivity	APL	-0.73	0.0049	0.038
Water temperature	APL	-0.82	5.30E-04	0.0083
Water temperature	ADS	-0.93	3.00E-06	2.30E-04
Daylight hours	AUP	-0.76	0.0041	0.036
Daylight hours	APL	-0.68	0.011	0.059
Daylight hours	ADS	-0.7	0.0082	0.058
Turbidity	APL	0.68	0.01	0.059
Turbidity	ADS	0.9	2.60E-05	0.001

Considering AGS further informs the identification of unusual samples. Both of the samples identified as unusual by k-mer composition clustering also have AGS values that are distinct from the other samples from their sampling sites. The September AUP sample has an AGS of 2.0 Mbp compared to the other AUP samples, which have a median of 3.0 ± 0.5 Mbp. The October sample from PDS has a high AGS of 3.7 Mbp compared to the site’s median of 2.4 ± 0.4 Mbp. The other October sample from the protected watershed (from PUP) also has an unusually high AGS (4.8 Mbp) compared with the other samples from that site (median = 3.1 ± 0.5 Mbp). This shared trend between the October PUP and PDS samples is unusual because they were collected 4 h apart from sites separated by a reservoir and a 9 km pipe. This may indicate that a system-wide change occurred that introduced higher-AGS bacteria, such as an extreme runoff event, or that these samples were similarly contaminated during or after sample collection.

The relationship between AGS and environmental conditions emphasizes the importance of considering AGS when analyzing metagenomic data. Beyond the direct biological relevance of AGS variability over time, this variation also indirectly affects gene functional group abundance profiles (Beszteri et al., 2010; Frank and Sørensen, 2011). This is because with lower AGS, universal, single-copy genes make up a larger proportion of functional genes, and vice-versa. This causes physically real yet biologically uninteresting variability in the abundance of universal, single-copy genes among samples with varying AGS, and introduces spurious correlations (Beszteri et al., 2010). In cases, such as the one seen here, where there is not only a large range of AGS values but also a strong relationship between AGS and environmental conditions, normalizing by AGS is important to avoid biased data interpretation. Previous studies have shown that AGS varies among environmental sampling sites (Angly et al., 2009; Frank and Sørensen, 2011) and within hosts (Nayfach and Pollard, 2015). To our knowledge, this work is the first to have tested for AGS temporal variation within sampling sites in environmental microbiomes. If not corrected for, this variation has the potential to obscure relationships between bacterial communities and environmental conditions, as described in the following section.

### Common Normalization Strategies Can Result in Contradictory Interpretations of Metagenomic Data

**FIGURE 4:**
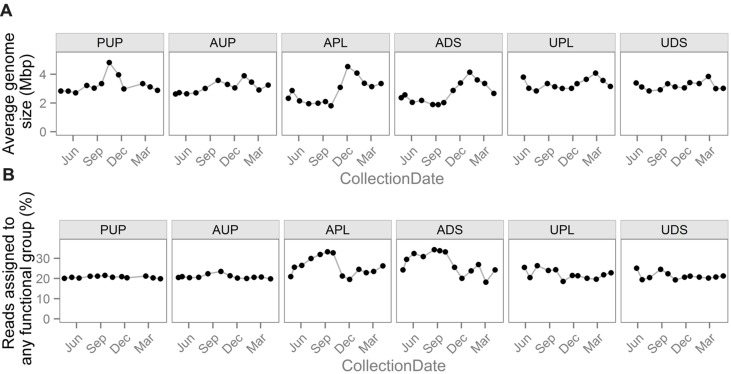
**Normalization factors used in different strategies to enable comparisons of gene group abundances between samples**. Notable differences exist among sites and over time in both **(A)** average genome size and **(B)** the percentage of reads assigned to any gene functional group. This variability demonstrates how normalization schemes using these values can have a drastic effect on downstream analyses.

To describe bacterial gene functional groups associated with land use, translated reads were compared to protein sequences with functional annotations and assigned functions based on sequence similarity. To compare the abundance of functional gene groups across samples, abundances must be normalized by “sequencing effort” (Chistoserdovai, 2010). Common normalization schemes involve one or more of the following components: (1) subsampling the data such that each sample has the same number of reads before assigning reads to functional groups, (2) dividing the number of reads assigned to each functional group by the total number of reads in a sample, (3) dividing the number of reads assigned to each functional group by the total number of reads assigned to all functional groups in a sample, that is, to normalize by the percentage of reads assigned, and (4) scaling by the number of cells represented by the reads. This last approach can involve multiplying by the AGS, which is estimated based on the abundance of multiple single copy genes (Angly et al., 2009; Manor and Borenstein, 2015; Nayfach and Pollard, 2015) or dividing by the abundance of a single copy gene. Normalization components 1 and 2 tend to give similar results except when considering low abundance groups, when they can result in the misinterpretation of zero counts, while components 3 and 4 can change results drastically (**Figure 4**).

In general, normalizing by the percentage of reads assigned is most commonly applied; however, this can lead to biases due to variation in read mappability (Manor and Borenstein, 2015). A read’s mappability to functional annotation databases can vary with technical differences, such as read-length, or with biological differences. For example, a read might not be assigned a function because it came from DNA that has an unknown function, that has diverged too much relative to reference sequences, or that is non-functional. If biological differences among metagenomes resulted in different percentages of reads assigned, then normalizing by total assigned reads per sample masks a real change in gene proportions. For example, this is likely to be the case if a community undergoes a shift from more well-characterized bacteria to more poorly characterized bacteria, when more and fewer reads will be functionally assigned, respectively. As certain phylogenetic branches of bacteria are better characterized than others, normalizing by the percentage of reads assigned can introduce bias.

Another biological factor that can affect the percentage of reads assigned is a change in AGS. In general, essential, core genes make up higher proportions of smaller genomes and are more likely to have a close homolog in the reference database, while larger genomes are more likely to contain more specialized genes that are less likely to have functionally characterized reference sequences (Raes et al., 2007; Nayfach and Pollard, 2015). This relationship is seen in the agriculturally affected sites (APL and ADS), where there is large variation in the percentage of reads assigned (**Figure 4B**) that is significantly negatively correlated with AGS (*r* = –0.74, –0.82; *p* = 0.005, 0.0009 for APL and ADS, respectively). This indicates that a biological shift has occurred and that normalizing functional profiles by the percentage of reads assigned would introduce bias. This relationship is not seen in the other sites, possibly due to the smaller ranges of AGSs or an uncharacterized confounding biological relationship.

Data normalization can lead to contradictory results. To illustrate this effect, we compared the abundance of level-two SEED functional groups between samples from the agriculturally affected sites (APL and ADS) collected in the “summer” period versus the “winter” period (**Figure 1**). Data was normalized in one of four ways: (1) only by even subsampling or by even subsampling followed by normalizing by: (2) the percentage of reads assigned, (3) AGS, or (4) the percentage of reads assigned and AGS. Out of 82 groups tested, 28 have differential abundances under all normalizations, three have differential abundances under only one normalization scheme, and 51 have differential abundances under two or three normalization schemes. Of those 28 functional groups with different abundances under all normalizations, 13 have opposite trends depending on the normalization used. For example, when abundance profiles are normalized by subsampling and AGS, the “Pathogenicity islands” functional category is more abundant in the rainy “winter” samples than the dry “summer” samples (fold change between medians = 1.1, *p* = 0.02, *q* = 0.03). When normalized in any of the other ways listed above, the relationship was opposite (fold changes = –1.4, –1.1, –1.1; *p* = 8*e*–5, 0.05, 0.02, 0.05; *q* = 0.0003, 0.08, 0.03, 0.08, for methods 1, 2, and 4 listed above, respectively). Though the scale of these differences is small, they are statistically significant and, in this example, directly relevant to water quality assessment. If this category of “Pathogenicity island” genes was targeted as a source of biomarkers in the development of a new water quality test, the choice of normalization scheme could directly affect whether a group of samples were considered high or low quality.

In theory, differential groups with larger effect sizes should be more robust to varying normalization methods. We see that here, where the groups with significant differences that agree across normalization methods have a greater average fold change than those that do not agree between normalizations (2.2 ± 0.6 versus 1.2 ± 0.2, respectively, Wilcox test *p* < 2.2*e*–16). This demonstrates that when looking for patterns in the abundances of functional groups among samples, the most conservative approach is to look for trends that are robust to normalization method. Given the potentially extreme differences that normalization methods can produce, the decision of which normalization steps to take should be chosen carefully and stated explicitly.

### Severity of Contamination in an Agriculturally Affected Watershed is Reflected in Gene Functional Group Abundances Across Sampling Sites

The Canadian Council of Ministers of the Environment (CCME) Water Quality Index (WQI) is a framework to evaluate surface water quality for the protection of aquatic life (Canadian Council of Ministers of the Environment, 2007). All samples from the sites not affected by agricultural pollution had a “good” or “excellent” water quality rating based on guidelines for ammonia, chloride, dissolved oxygen, nitrate, pH, and orthophosphate. This includes the urban samples, indicating that either (1) land use did not have as large an impact on these samples as it did on the agriculturally affected samples, or (2) that this index formulation is not appropriate to assess their water quality. In the agriculturally affected sites, samples from the drier months (May to October) had CCME WQI ratings of “fair” or “good” quality, while samples from the wetter months (November to April) all had a “marginal” or “poor” rating, due to high orthophosphate and/or low dissolved oxygen concentrations.

**FIGURE 5:**
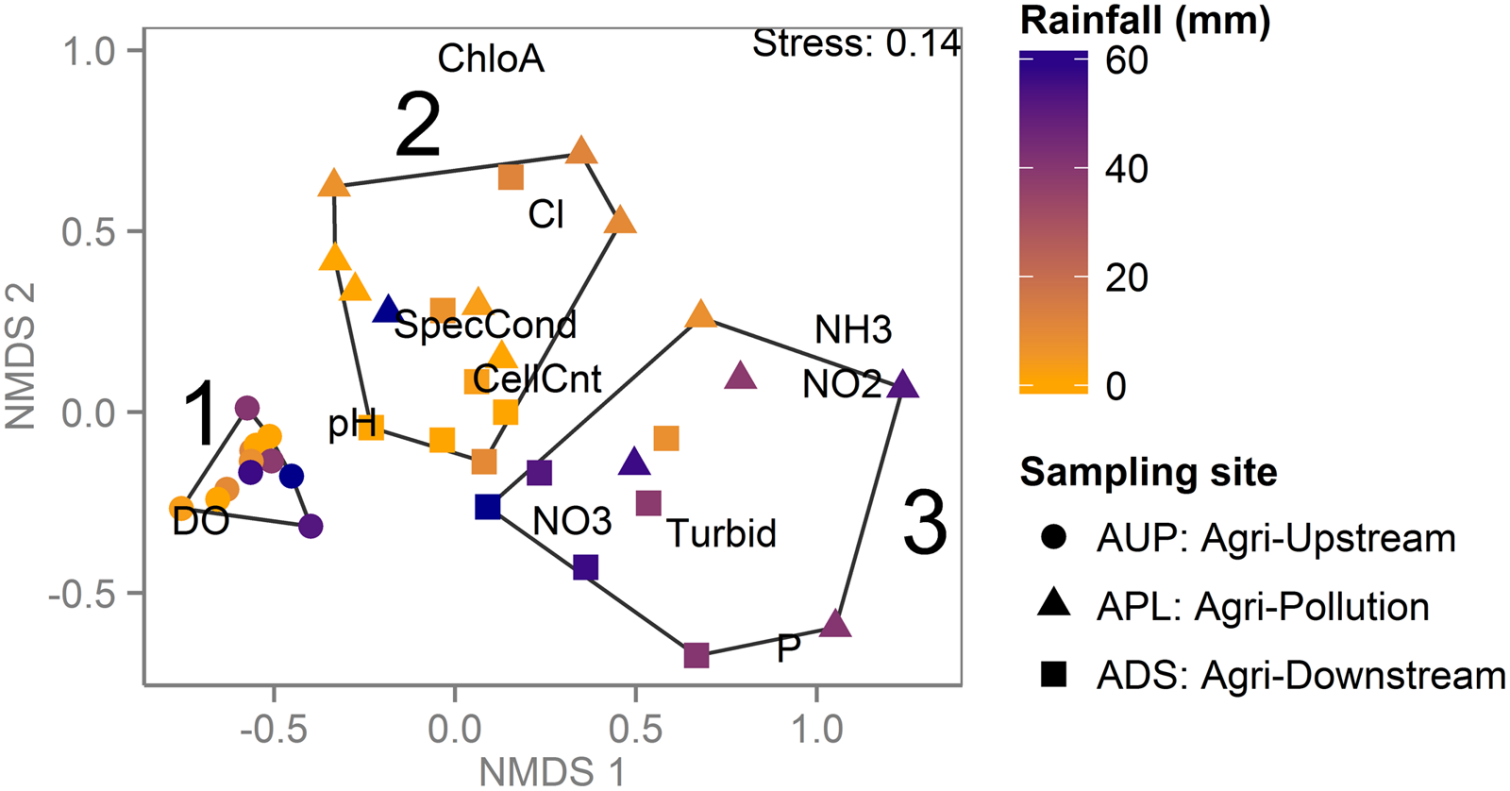
**Agricultural watershed samples clustered by water chemistry reveal impact of land use and rainfall**. NMDS plot based on environmental and chemical water measurements for samples from the agricultural watershed. Each point represents a sample, colored by cumulative rainfall over 3 days prior to sampling and shaped by sampling site. Significant clusters are outlined and numbered in black. Samples collected upstream of agricultural activity (Cluster 1) have higher DO levels. Samples collected in the summer from the agriculturally affected sites (Cluster 2) have higher chlorophyll *a* concentration, while the winter samples are more affected by runoff, as indicated by higher nutrient levels and turbidity (Cluster 3).

A similar classification was seen when samples from the agricultural watershed were clustered based on water chemistry and biological measures indicative of contamination (**Figure 5**). Three high-level clusters are significant (bootstrap value > 90%); Cluster 1 comprised samples from the unaffected site, while Clusters 2 and 3 contained a mix of samples from both affected sites. Consistent with k-mer clustering patterns and WQI ratings, Cluster 2 is mostly composed of samples collected in the drier months (May to October), while Cluster 3 is mostly from samples collected in the rainier months (November to April). The only exception to this pattern is the December sample from APL, which has very low values of ammonia, nitrate and nitrite and is grouped in Cluster 2. The two samples with lower rainfall in Cluster 3 were collected in early February, when rainfall was below seasonal averages. The rainy season samples from the agriculturally affected sites (Cluster 3) are associated with elevated turbidity and higher concentrations of nitrate, nitrite, ammonia, and orthophosphate, consistent with increased runoff from the surrounding agricultural land (**Figure 5**).

This increased runoff and agricultural impact is reflected in the metagenomic data when SEED subsystem abundances are compared between the more affected samples (Cluster 3) and the less affected samples (Clusters 1 and 2). When abundances are normalized by even subsampling and AGS, 191 groups are significantly more abundant in the more affected samples (**Supplementary Table S1**). When only considering the 11 groups with larger effect sizes (fold change between medians ≥2.5; **Table 4**), these groups are significantly different under all normalization conditions (normalized by subsampling only, by the percentage of reads assigned, and by the percentage of reads assigned and AGS). When considering more subtle differences (fold change <2.5), 93 groups are only predicted as significantly different when normalized by even subsampling and AGS. In general, we see a higher abundance of subsystems associated with nutrient metabolism (carbohydrates, nitrogen, and proteins), respiration, and phage in those samples that had increased runoff from agricultural land (Cluster 3; **Table 4**, **Supplementary Table S1**). The higher abundance of nutrient-metabolism subsystems is reasonable, given the higher concentrations of nitrate, nitrite, ammonia, and orthophosphate (**Figure 5**). As this data describes DNA extracted from material collected on 0.2 μm pore-size filters, most of the phage sequences are likely associated with cells as prophage, replicating phage, or phage particles attached to the surface of cells. Further study of bacterial taxonomic profiles and these specific gene sequences may provide insight into whether this SEED subsystem is more abundant due to changes in host abundance or viral population dynamics. This analysis is limited by the biases inherent in how metagenomic reads are classified, in gene lengths, and in the SEED classification structure. However, the differential subsystems with larger effect sizes indicate that there are significant differences in the genes present between water samples with lower water quality, collected during a period of increased runoff from agricultural land, and samples with higher water quality, collected from the same agricultural area but during a dryer period.

**Table 4:** Differentially abundant gene functional groups between samples with higher and lower water quality in the agricultural watershed.

Differential SEED subsystem	SEED class (Subclass)	*q*-value^1^	Fold change^2^
Malonate decarboxylase	Carbohydrates (Organic acids)	0.0045	4.6
Nitrosative stress	Nitrogen metabolism (No subclass)	0.0045	3.7
Denitrification	Nitrogen metabolism (No subclass)	0.0065	3.5
Phage capsid proteins	Phages, prophages, transposable elements (Bacteriophage structural proteins)	0.0045	3.5
Na(+)-translocating NADH-quinone oxidoreductase and rnf-like group of electron transport complexes	Respiration (Electron donating reactions)	0.0074	2.9
Lysine degradation	Amino acids and derivatives (Lysine, threonine, methionine, and cysteine)	0.012	2.8
Pyruvate:ferredoxin oxidoreductase	Carbohydrates (Central carbohydrate metabolism)	0.0065	2.8
Bacterial hemoglobins	Stress response (No subclass)	0.013	2.7
d-galactarate, d-glucarate, and d-glycerate catabolism	Carbohydrates (Monosaccharides)	0.0065	2.6
d-galactonate catabolism	Carbohydrates (Monosaccharides)	0.0098	2.6
Pyrimidine utilization	Nucleosides and nucleotides (Pyrimidines)	0.0065	2.6
RNA 3′ terminal phosphate cyclase	RNA metabolism (RNA processing and modification)	0.0065	2.5

*Lower-quality water samples are more affected by agricultural runoff than the higher-quality samples (Cluster 3 versus 1 and 2 in **Figure 5**). Differential functional groups (SEED subsystems) with fold change greater than 2.5 are listed and sorted by fold change. Abundances are normalized by AGS. Most of the subsystems that are more abundant in the lower-quality water are responsible for metabolism of nutrients, consistent with the water’s higher concentrations of nitrate, ammonia, and orthophosphate.*

*^1^Corrected for FDR. ^2^Median abundance in lower quality water divided by median abundance in higher quality water*.

### Gene Group Proportions Normalized by Percentage of Reads Assigned are Stable Across Time and Watershed but Differ from Previous Studies

When SEED level 2 subsystem abundances are normalized across samples by the percentage of reads assigned to any subsystem, the median standard deviation of abundances is 0.2 ± 0.1% among the 35 subsystems with at least 1% abundance, which corresponds to a 10 ± 6% median relative standard deviation (i.e., standard deviation calculated as relative to subsystem abundance). This low variability when normalizing by percentage of reads assigned was also seen in the only other metagenomic study that has looked at gene family composition in river water across land use (Staley et al., 2014b). In this study, high-level KEGG pathway category abundances were remarkably stable in the Upper Mississippi across varying land use (forested, urban, and agricultural). Across 11 sites they observed a maximum standard deviation of 0.5% among KEGG level 2 groups, with a median standard deviation of 0.02%. However, this study only sampled each site once (between May to July), whereas, we sampled monthly over a year, and they used MG-RAST to assign reads to functional groups, whereas we used MEGAN.

In order to better compare our results against this Upper Mississippi study, we analyzed our data 1 month at a time using their methods. Briefly, we used the MG-RAST pipeline with KEGG database annotations and normalized by the percentage of reads assigned, without adjustments for AGS. This analysis excluded the non-surface sampling site, PDS, and is limited by the normalization choices and the accuracy of MG-RAST. To match the single time point samples of the Upper Mississippi study, we examined our May to July samples by month. In this analysis, we saw higher variability within KEGG level 2 groups, with a maximum standard deviation of 2% and a median standard deviation of 0.2%, versus 0.5% and 0.02% in the Upper Mississippi study, respectively. This higher variability in our study when looking over the same time period may indicate that the land use differences between sites in this study are greater than those in the Upper Mississippi study or may be due to inherent differences in the watersheds. In both cases, however, when looking at functional assignments as a proportion of all reads assigned a function (i.e., when normalized by the percentage of reads assigned) the variability in abundance of these high-level functional groups is low compared to their abundance values. This demonstrates how normalizing by the percentage of reads assigned and failing to normalize by AGS can mask variability among samples. When the patterns of these potential normalization factors are considered, there is clear variability among samples (**Figure 4**).

Though the variability among high-level functional groups analyzed this way was similar between our study and the Upper Mississippi study, notable differences were observed between which groups were most abundant. According to this MG-RAST analysis performed for comparative purposes and lacking AGS normalization, the most abundant KEGG level 2 groups across all sites in our data were “Amino Acid Metabolism” (20 ± 2%), “Carbohydrate Metabolism” (12 ± 0.9%) and “Membrane Transport” (11 ± 1%), while in the Upper Mississippi study, the most abundant categories were “Membrane Transport” (21 ± 0.3%), “Carbohydrate Metabolism” (11 ± 0.1%) and “Signaling molecules and interaction” (11 ± 0.3%). The differences in abundance of “Amino Acid Metabolism” and “Signaling molecules and interaction pathways” were especially pronounced, with 20% versus <5% and <0.06% versus 11% abundance in our study versus the Upper Mississippi study, respectively. These differences in dominant functional groups may be due to technical differences, such as our longer read lengths and different filtration system, or also may suggest that functional profiles differ over large distances. This highlights the inherent difficulty in comparing metagenomics analyses across different studies at present and at minimum the need to consider methodology variation. Regardless, these considerable inter-study differences are notable since there is so little within-study variation when analyzed in this way. Our results from three watersheds that were all measured using the same methodology, but are up to 130 km apart and under differing land use suggest that, among functionally assigned reads not adjusted for AGS, general functional profiles are fairly stable at a regional scale. Among AGS-adjusted profiles not calculated as proportions of functionally assigned reads, however, we do see more variation, both between sites and within sites over time. Further studies, in which variability in AGS and in the proportions of reads assigned are considered, are required to characterize the variability of river metagenomes at larger geographic scales.

## Conclusion

This work presents the first year-long survey of bacterial metagenomes from water samples collected across protected, agricultural, and urban watersheds. It is also the first to report metagenome gene functional group differences associated with land use across time in lotic microbiomes. We have shown that fundamental metagenome characteristics such as k-mer composition and AGS vary with time and land use. Sampling site can be the major discriminative factor in metagenome k-mer composition when site characteristics are very different (i.e., water collected from a reservoir through a pipe versus surface water); however, among samples of surface water, metagenomes instead clustered by water chemistry, even when collected from unconnected watersheds 130 km apart. AGS is correlated with hours of daylight in all sites in the agricultural watershed. Beyond its ecological relevance, this finding also demonstrates the importance of normalizing functional profiles by AGS, since this variation could confound relationships between metagenomes and environmental conditions. Further bias can be introduced by the common practice of normalizing gene prediction abundances by the number of predictions made. When comparing samples with differing water quality, many gene functional groups with differential abundance were observed and those with the largest effect sizes were robust to normalization method. However, when considering more subtle effects, normalization strategy can have a large impact on both the interpretation of gene functional group profiles and also the identification of biomarkers. This study has shown that metagenome characteristics and gene function content change with time and land use in lotic bacterial communities. Future studies may build on the results presented here either directly, through the identification of candidate biomarkers from the sequence data presented, or indirectly, by using the findings reported as a reference in the design of future studies of freshwater ecosystem health.

## Author Contributions

TV led the bioinformatics analyses and wrote the manuscript. MP contributed to analyses and revised the manuscript. MU-D led the sampling and DNA sequencing, with help from MC and KC. JS and MN performed size selection of sequencing libraries. CS and WH guided the analyses and aided in interpretations. PT, NP, and FB designed the project, guided the analyses and aided in interpretations, with FB also providing lead guidance in manuscript revisions. All authors contributed to final revisions of the manuscript.

## Conflict of Interest Statement

JS and MN both hold shares in Coastal Genomics, a privately owned company that provided the Ranger Technology used in this study. The other authors declare that the research was conducted in the absence of any commercial or financial relationships that could be construed as a potential conflict of interest.

## Supplementary Material

TABLE S1 **| Differentially abundant gene functional groups between samples with higher and lower water quality in the agricultural watershed**.
